# One for two, ipsilateral reduction and contralateral reconstruction mammoplasty: A case report

**DOI:** 10.1016/j.ijscr.2020.06.075

**Published:** 2020-06-22

**Authors:** Abdulwahid M. Salih, Zuhair D. Hammood, Fahmi H. Kakamad, Karzan M. Salih, Hiwa O. Baba, Hunar A. Hassan, Shvan H. Mohammed, Goran A. Qadir, Hemn A. Hassan, Ismael Y. Abdullah

**Affiliations:** a Faculty of Medical Sciences, School of Medicine, Department General Surgery, University of Sulaimani, Sulaimani, Kurdistan, Iraq; b Smart Health Tower, François Mitterrand Street, Sulaimani, Kurdistan, Iraq; c Kscien Organization, Hamdi Str, Azadi Mall, Sulaimani, Kurdistan, Iraq; d Iraqi Board for Medical Specialties, Department of General Surgery, Sulaimani Center, Kurdistan, Iraq; e College of Science, Department of Biology, University of Sulaimani, Kurdistan, Iraq

**Keywords:** Mammoplasty, Reduction, Flap, Case report

## Abstract

**Introduction:**

The aim of this paper is to introduce a novel procedure for concomitant contralateral reduction and ipsilateral reconstruction mammoplasty by dividing the contralateral breast and creating a pectoralis myocutaneous flap for reconstruction.

**Presentation of case:**

A 34-year-old female, with a history of left side mastectomy presented for reconstruction. Under general anesthesia, the scar of the previous operation was resected in an elliptical shaped incision, the right breast was divided in middle, leaving the flap (pectoralis myocutaneous flap) with pectoralis branch of thoracoacromial artery. The superiomedial part of right breast (the flap) was rotated under the bridge of intermammary skin into the left incision. Ten days after the operation, the flap was viable and healthy.

**Discussion:**

The aim of breast reconstruction is to provide psychosocial support and improve quality of life in the long term by restoring the shape of the breast surgically. New techniques have emerged constantly, and each comes with its list of advantages and risks. The benefits of reconstruction are clear, yet often it is not found as a part of breast cancer treatment routinely.

**Conclusion:**

In single session reduction mammoplasty in one breast and using the resected piece as a flap to reconstruct the contralateral breast is possible whenever indication.

## Highlights

• There is a considerable debate regarding implant or autologous reconstruction of breast.
• Single session reduction mammoplasty with contralateral autologous reconstruction is feasible.
• A novel procedure has been presented.
• Dividing the contralateral breast and creating a myocutaneous flap for reconstruction.

## 1 Introduction

Breast cancer is a worldwide problem with 1.7 million newly diagnosed cases each year. Drastically up to 40% of all these cases require mastectomy as a part of their surgical treatment [1]. Among the diagnosed cases, (77.3%) are women of age between 40 and 49 [2]. Mastectomy is often a turning point in a patient’s life affecting self-esteem and body image leading to sexual problems and overall quality of life [3]. There has been a steady increase in patient’s receiving breast reconstruction after mastectomy, especially after 1998, where breast reconstruction coverage by all-payer insurances was mandated by Women’s Health and Cancer Rights Act [4].

There is a considerable debate regarding implant or autologous reconstruction of breast and whether one of them is superior. There are numerous studies advocating one over the others [5].

In this paper, a novel procedure has been introduced for concomitant contralateral reduction and ipsilateral reconstruction mammoplasty by dividing the contralateral breast and creating a pectoralis myocutaneous flap for reconstruction.

The work has been reported in line with SCARE guidelines [6].

### 1.1 Patient information

A 34-year-old, married, non-smoker female, with history of invasive ductal carcinoma of left breast treated with mastectomy and axillary lymph node dissection before two years presented for reconstruction. She completed her six cycles of chemotherapy. Her past medical, past surgical, drug and family histories were clear.

### 1.2 Clinical findings

**Fig. 1:**
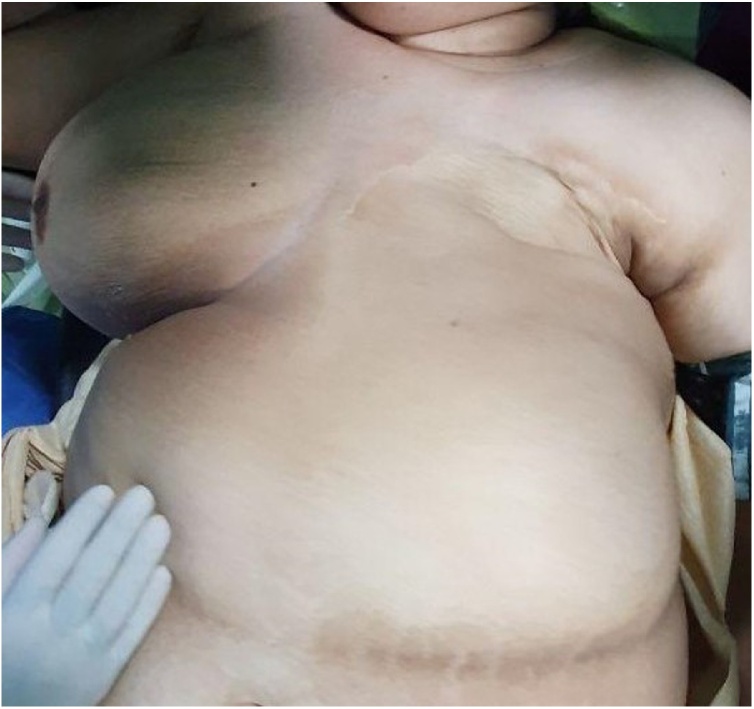
Preoperative photo showing large right breast with absence of left breast.

On examination, she was an obese (body mass index 31 kg/m^2^) patient with an old scar in the site of left breast and a large pendular breast on the right site (Fig. 1).

### 1.3 Therapeutic intervention

**Fig. 2:**
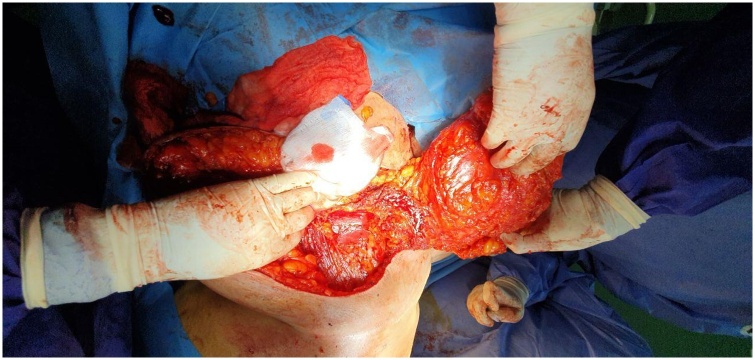
Intraoperative picture showing flap elevation.

**Fig. 3:**
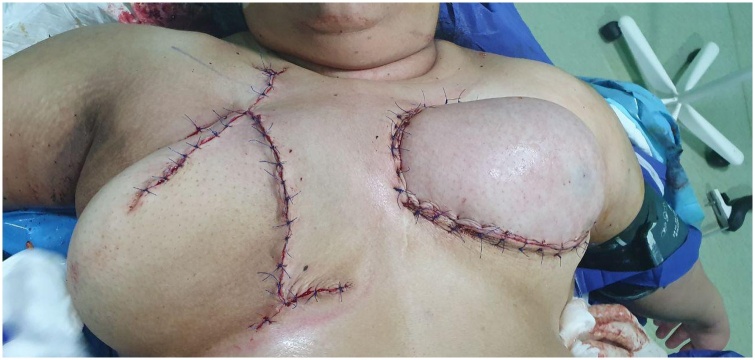
End of the operation, wound closure.

The patient was prepared for general anesthesia, in supine position, the scar of the previous operation was resected in an elliptical shaped incision, right breast was divided in middle in an elliptical form, leaving the flap (pectoralis myocutaneous flap) with pectoralis branch of thoracoacromial artery. The superiomedial part of right breast (the flap) was elevated, and rotated under the bridge of intermammary skin into the left incision in the position of the previously removed left breast (Fig. 2). Two drains were put and the incisions were closed in layers after resection of the skin ears (Fig. 3). The operation was performed by the first author with assistance of the second, fourth and fifth authors.

### 1.4 Follow-up and outcomes

**Fig. 4:**
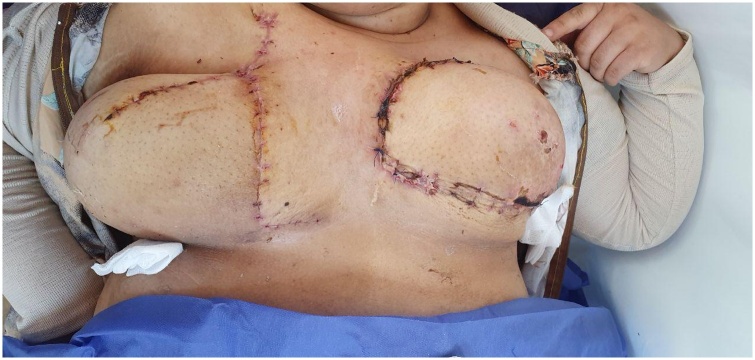
Viable flap ten days after the operation.

The post-operative course was uneventful. The patient remained in hospital overnight and she was sent home on the first operative day. Ten days after the operation, the flap was viable and healthy (Fig. 4). Unfortunately, she was lost from follow up later.

## 2 Discussion

Advancement in breast reconstruction continuously aims to provide a more natural breast outcome in terms of shape, texture and symmetry that is in line with the other side, whilst keeping complications risk to minimum, all in order to improve the quality of life [7].

The aim of breast reconstruction is to provide psychosocial support and improve quality of life in the long term by restoring the shape of the breast surgically [3]. New techniques have emerged constantly, and each comes with its list of advantages and risk [8]. The benefits of reconstruction are clear, yet often it is not found part of breast cancer treatment routinely [3].

Educating patients about reconstruction options before their surgery allows for a better-informed decision-making and decreases decision regret after reconstruction procedures. This is emphasized in Swedish guidelines for care and treatment of breast cancer patients [9]. As such, surgeons are required to have extensive knowledge regarding possible complications and their contributing factors to allow for proper counseling of the patient and guide the decision making as a shared process [10]. Five common techniques are widely used for breast construction: Expander/implant, Latissimus Dorsi myocutaneous (LD)-flap, LD flap combined with an implant, Deep Inferior Epigastric Perforator (DIEP) flap and free Transverse Rectus Abdominis Musculocutaneous (TRAM)-flap [11].

Autogenous breast reconstruction has seen new developments and techniques in recent years that yield a favorable functional outcome, however aesthetic appearance is significantly influenced by amount and quality of retained tissue after mastectomy. There seems to be insufficient details regarding factors that influence the type of mastectomy and the subsequent design of autogenous skin paddle, despite their implicit role in breast reconstruction [12]. Although still not standardized, pectoral muscle flaps have been commonly used in the last three decades. There are important debatable technical points for this technique like release of fascia, extended mobilization, muscle fiber splitting and preservation of thoracoacromial vessels, perforator vessels and humeral insertion [13]. There has been thorough discussion for the favorable outcomes for pectoralis major muscle flaps. Thoracoacromial artery is the main supply and is unaffected when internal mammary arteries are harvested for coronary grafting [14].

The current approach is aimed to release pectoralis major from the site of humeral insertion and to be used as an advancement flap. While maintaining the thoracoacromial vessels, the intercostal perforators are to be sacrificed aiming to maximize muscle perfusion and bulk and minimize functional impairment. The use of a combination of pectoral muscle and contralateral breast tissue could be an optimal autologous breast reconstruction tissue strategy.

In single session reduction mammoplasty in one breast and using the resected piece as a flap to reconstruct the contralateral breast is possible whenever indication. The indication might be absence of one breast and very large contralateral breast, in other words, requirement of both reduction and reconstruction mammoplasty. This strategy (two operations in single session) reduces operative time, decreases the number of general anesthesia, avoids foreign body implantation and provides the removed breast with natural breast tissue.

## Sources of funding

No source to be stated.

## Ethical approval

Approval is not necessary for case report in our locality.

## Consent

Consent has been taken from the patient and the family of the patient.

## Author contribution

Abdulwahid M.Salih, Zuhair D.Hammood: Surgeons performing the operation, writing and final approval of the manuscript and follow up.

Fahmi H.Kakamad, Karzan M.Salih, Shvan H.Mohammed^3^, Goran A.Qadir: Writing the manuscript, final approval of the manuscript.

Hiwa O.Baba, Hunar A.Hassan Hemn A.Hassan, Ismael Y. Abdullah: literature review, final approval of the manuscript.

## Registration of research studies

Not applicable.

## Guarantor

Fahmi Hussein Kakamad.

## Provenance and peer review

Not commissioned, externally peer-reviewed

## Declaration of Competing Interest

There is no conflict to be declared.
